# The Effect of FATP1 on Adipocyte Differentiation in Qinchuan Beef Cattle

**DOI:** 10.3390/ani11102789

**Published:** 2021-09-24

**Authors:** Xuchun Liu, Shijun Li, Liyun Wang, Weiyi Zhang, Yujuan Wang, Linsheng Gui, Linsen Zan, Chunping Zhao

**Affiliations:** 1 College of Animal Science and Technology, Northwest A&F University, No. 22 Xinong Road, Yangling 712100, China; 13210925027@nwafu.edu.cn (X.L.); sjli3@gzu.edu.cn (S.L.); wangliyun_2019@nwafu.edu.cn (L.W.); 2017050395@nwafu.edu.cn (W.Z.); 2017050406@nwafu.edu.cn (Y.W.); zanls2011@nwafu.edu.cn (L.Z.); 2 College of Agriculture and Animal Husbandry, Qinghai University, Xining 810016, China; 2017990039@qhu.edu.cn

**Keywords:** FATP1, bovine adipocyte, overexpression, interference, RNA-seq

## Abstract

**Simple Summary:**

Previous research found that FATP1 plays an important role in the regulation of fatty acid metabolism and lipid accumulation in pig and chicken, but its function has not been explored in bovine adipocyte yet. In this study, we investigated the effect of *FATP1* expression on preadipocyte differentiation in Qinchuan cattle using overexpression and interference assays. Our results reveal that *FATP1* overexpression promoted preadipocyte differentiation, lipid droplet formation, and the expression of *LPL* and *PPARγ*, while *FATP1* interference had the opposite effects on adipocyte differentiation and fat deposition. Following *FATP1* overexpression and *FATP1* interference in adipocytes, RNA-seq analysis identified that *SLPI*, *STC1*, *SEMA6A*, *TNFRSF19*, *SLN*, *PTGS2*, *ADCYP1*, *FADS2*, and *SCD* genes were differentially expressed. Pathway analysis revealed that the PPAR signaling pathway, AMPK signal pathway, and Insulin signaling pathway were enriched with differentially expressed genes. We propose that the *FATP1* gene may affect the beef quality by involving adipocyte differentiation and lipid deposition, and may shed new light on the formation mechanisms of adipose tissues.

**Abstract:**

FATP1 plays an important role in the regulation of fatty acid metabolism and lipid accumulation. In this study, we investigated the patterns of *FATP1* expression in various tissues obtained from calf and adult Qinchuan cattle, and in differentiating adipocytes. Next, we investigated the effect of *FATP1* expression on preadipocyte differentiation in Qinchuan cattle using overexpression and interference assays. We also identified the differentially expressed genes (DEGs) and pathways associated with *FATP1* overexpression/interference. Our results reveal that *FATP1* was broadly expressed in heart, kidney, muscle, small intestine, large intestine, and perirenal fat tissues. While *FATP1* overexpression promoted preadipocyte differentiation, fat deposition, and the expression of several genes involved in fat metabolism, *FATP1* interference had the opposite effects on adipocyte differentiation. Following *FATP1* overexpression and *FATP1* interference in adipocytes, RNA-seq analysis was performed to identify DEGs related to fat metabolism. The DEGs identified include *SLPI*, *STC1*, *SEMA6A*, *TNFRSF19*, *SLN*, *PTGS2*, *ADCYP1*, *FADS2*, and *SCD*. Pathway analysis revealed that the DEGs were enriched in the PPAR signaling pathway, AMPK signal pathway, and Insulin signaling pathway. Our results provide an in-depth understanding of the function and regulation mechanism of FAPT1 in fat metabolism.

## 1. Introduction

Fatty acid transport proteins (FATPs) are evolutionarily conserved transmembrane proteins in the fatty acid transport family. FATPs facilitate long-chain fatty acids (LCFA) importing into the cell by translocation at the plasma membrane [1]. In mammals, six different isoforms of FATPs (FATP1–FATP6) showing tissue-specific expression patterns have been identified as playing crucial roles in fatty acid metabolism [2]. FATP1, also known as Solute Carrier Family 27 Member 1 (SLC27A1), was the first reported family member, and is highly expressed in muscle, fat, heart, and liver [3,4], which are all characterized by high fatty acid uptake and rapid fatty acid metabolism. FATP1 may promote LCFA circulation flux. In addition, FATP1 may influence fatty acid metabolism and lipid accumulation by coordinating fatty acid esterification and oxidation [2].

The function and mechanism of action of FATP1 in fatty acid metabolism have been extensively researched in yeast and other cells. In *S. cerevisiae*, knockout of *FATP1* decreased the activities of long chain acyl CoA synthetases [5]. In yeast, overexpression of *FATP1* promoted an 8.2-fold increase in the fatty acid transport rate [6]. In 293 cells, FATP1 exerted overall control of triglyceride biosynthesis, and overexpression of *FATP1* enhanced fatty acid uptake and increased triglyceride content [7]. In 3T3-L1 adipocytes, knockdown of *FATP1* decreased triglyceride accumulation and reduced droplet size [8].

The role of FATP1 in fat deposition and adipocyte differentiation in livestock has also been widely researched. A positive correlation between FATP1 and intramuscular fat deposition has been reported in pig [9]. Moreover, overexpression of *FATP1* induced intramuscular preadipocyte proliferation and fat accumulation in pig [10]. *FATP1* overexpression also significantly upregulated the expression levels of PPARγ, CEBPα, LPL, PLIN1, and FASN, and downregulated the expression of β-catenin. It was subsequently hypothesized that FATP1 might repress the Wnt,/β-catenin signaling pathway and stimulate adipogenesis in porcine intramuscular preadipocytes [10]. This hypothesis is supported by the results of RNA interference experiments in chicken. Thus, silencing of *FATP1* inhibited chicken preadipocyte differentiation and decreased the expression levels of FAS, C/EBPα, and PPARγ in chicken preadipocytes [11]. *FATP1* expression levels were also positively correlated with intramuscular fat content in Korean cattle [12]. However, the corresponding mechanism of action has not been further explored in cattle using loss-of-function or gain-of-function experiments.

The bovine *FATP1* gene is located on Chromosome 7, and comprised of 14 exons and 13 introns extending over more than 40 kb of genomic DNA [13]. In the present study, we analyzed *FATP1* expression patterns in the tissues and adipocytes of Qinchuan cattle. In addition, we explored the effects of FATP1 on preadipocyte differentiation using overexpression and interference assays. DEGs and related signal pathways were identified using RNA-sequencing and data analysis. Our findings help further understanding of the role of FATP1 in adipocyte differentiation and lipid metabolism in bovine.

## 2. Materials and Methods

### 2.1. Sample Collection

All animal procedures were approved by the Animal Care and Use Committee of Northwest A&F University (Yangling, China). Three adult male Qinchuan cattle and three newborn male calves were obtained from the National Beef Cattle Improvement Center Farm (Yangling, China). The cattle were humanely euthanized and the following tissues were harvested: heart, spleen, liver, lung, rumen, kidney, reticulum, omasum, small intestine, abomasum, fat, and longissimus dorsi muscle. The harvested samples were rinsed in 1 × PBS, transferred to sterile, DNase and RNase free tubes, snap-frozen in liquid nitrogen, and finally stored at −80 °C for further analysis.

### 2.2. Chemical Synthesis and Transfection of siRNA

Three complementary pairs of siRNA (siRNA1, siRNA2, siRNA3) oligos (designed to inhibit the expression of the bovine *FATP1* gene) and control siRNA (siNC) oligos were synthesized by Shanghai GenePharma Co., Ltd. (Shanghai, China). The specific siRNA sequences are shown in Table 1.

### 2.3. Construction of FATP1 Overexpression Adenovirus Vector

The CDS for bovine *FATP1* was first cloned into a T vector. After digestion with NheI and HindIII, a fragment encoding the *FATP1* CDS was then cloned into a pAd-Track shuttle vector. The shuttle vector encoding the *FATP1* CDS was subsequently digested with Pme1 enzyme (NEB, Ipswich, MA, USA) to facilitate recombination with the pAdEasy-1 vector. After confirmation of recombination, the recombinant vector was digested with PacI. The linearized vector was then purified, packaged into adenovirus (designed as Ad-*FATP1*,/Ad-NC), and transfected into HEK 293A cells.

### 2.4. Bovine Preadipocyte Isolation and Culture

Preadipocytes were isolated from newborn Qinchuan calves as follows. Under sterile conditions, perirenal adipose tissues were collected and washed three times using 1 × PBS containing 1% penicillin/streptomycin. The adipose tissue samples were then sheared into pieces, and two volumes of 1 mg/mL collagenase type I was added (Sigma, Shanghai, China). The samples were then digested with constant shaking at 37 °C for 90 min. After digestion, the mixture was filtered, and cell lysate was added to the filtrate. After centrifugation, the cells were seeded into cell culture dishes containing DMEM/F12 (Gibco, Waltham, MA USA) with 10% FBS (Invitrogen, San Diego, GA, USA) and antibiotics. The cells were then incubated at 37 °C in a cell culture incubator using 5% CO_2_.

### 2.5. Preadipocyte Differentiation, Transfection and Infection

Preadipocyte differentiation was induced using induction medium containing DMEM/F12, 10% FBS, 1% antibiotics, 5 μg/mL insulin (Sigma, St. Louis, MO, USA), 1 μm DXMS (Sigma), and 0.5 mm IBMX. After 48 h, preadipocyte differentiation was continued using induction maintenance medium containing DMEM/F12, 10% FBS, 5 μg/mL insulin, and 1% antibiotics. For *FATP1* knockdown, cells at 80% confluence were transfected by siRNAs or si-NC using a Lipofectin 3000 transfection kit (Invitrogen, San Diego, GA, USA) according to the manufacturer’s instructions. For overexpression of FATP1, Ad-*FATP1* or Ad-NC were used to infect cells at a multiplicity of infection (MOI) of 25.

### 2.6. Oil Red O Staining

After experimental treatment, cells were washed three times in 1 × PBS, and then fixed for 30 min with 4% paraformaldehyde. The cells were then stained using Oil red O staining solution for 30 min and washed with 1 × PBS. Finally, representative images were obtained using an Olympus IX71 microscope.

### 2.7. Total RNA Extraction and qRT-PCR Analysis

Total RNA was isolated using TRIzol^TM^ Reagent (Invitrogen, San Diego, GA, USA) according to the manufacturer’s instructions. RNA integrity and RNA concentration were analyzed by 1% agarose gel electrophoresis and UV spectrophotometry. cDNA was synthesized from total RNA using the Prime Script^TM^ RT Kit (Takara, Dalian, China). qRT-PCR was performed using the SYBR Green PCR Master Mix Kit (Takara, Biotech, Dalian, China) on a 7500 Real-Time PCR System (Applied Biosystems, Forster City, CA, USA) according to the manufacturer’s recommendations. qRT-PCR primer information is listed in Table 2. Target gene expression levels were analyzed by the 2^−ΔΔCt^ method using *ACTB* to normalize expression.

### 2.8. Total Protein Extraction and Western Blotting

Cells were lysed with RIPA (Beyotime, Haimen, China) containing protease inhibitor cocktail (Roche, Indianapolis, IN, USA). Total protein samples (20 µg per well) were resolved by a 12% SDS-PAGE gel. The resolved protein bands were then transferred to PVDF membranes. Next, the PVDF membranes were blocked with fat-free milk (5% *w*/*v*), and incubated with primary antibody overnight at 4 °C. The following primary antibodies were used: anti-FATP1 (ab 2759688 ABclonal, Woburn, MA, USA); anti-LPL (bs-2336R Bioss, Beijing, China); anti-FADS2 (bs-11516R Bioss, Beijing, China); and anti-SCD1 (ab236868 Abcam, Waltham, MA, USA). After washing three times with TBST, the membranes were incubated with secondary antibody for 2 h. Finally, the luminescence signals were detected using a ChemiDoc^TM^ XRS+ System (Bio-Rad, Hercules, CA, USA).

### 2.9. RNA Sequencing and Functional Enrichment Analysis of DEGs

Following pre-treatment with Ad-*FATP1*, Ad-NC, siRNA, and siNC, the preadipocytes (three replicates for each treatment) were induced to differentiate on the fourth day. In total, 12 cell samples (four treatments × three replicates) were harvested for subsequent analysis by RNA-Seq. RNA sequencing analysis was performed by Novogene. After processing of the RNA-Seq results, DEGs were identified using DESeq software. DEGs are defined as genes with |log_2_ (Fold Change)| ≥ 1.5 and adjusted *p* ≤ 0.05. Go enrichment analysis and KEGG enrichment analysis of DEGs were implemented by cluster Profiler (3.4.4) software. GO terms with a *p* value ≤ 0.05 were defined as significantly enriched.

### 2.10. Statistical Analysis

All data analysis was performed using SPSS 19.0. The data is presented as mean ± SD. The data were analyzed using a Dunnett’s multiple comparison test and one-way ANOVA. * *p* ≤ 0.05, ** *p* ≤ 0.01 were used to define statistical significance. All experiments were performed (at least) in triplicate.

## 3. Results

### 3.1. FATP1 Expression Patterns in Qinchuan Cattle Tissues and Differentiated Preadipocytes

**Figure 1:**
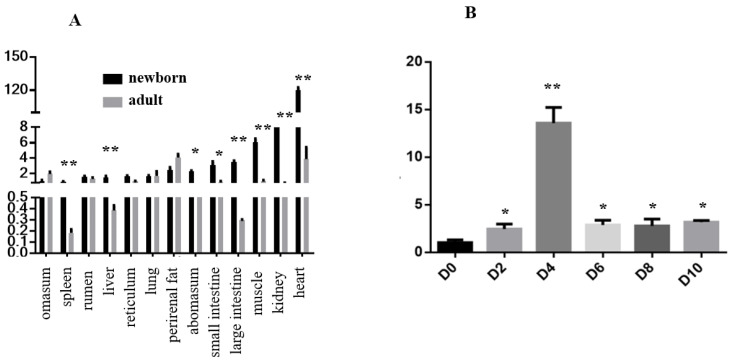
Expression of FATP1 in tissues and during preadipocyte differentiation in Qinchuan cattle. (**A**) FATP1 mRNA levels in tissues (*n* = 3). (**B**) FATP1 mRNA levels at day 0 (D0), day 2 (D2), day 4 (D4), day (D6), day 8 (D8) and day 10 (D10) after preadipocyte differentiation. * *p* ≤ 0.05, ** *p* ≤ 0.01 adult versus newborn (**A**) and D2 (D4, D6, D8, D10) versus D0 (**B**).

To explore the expression characteristics of *FATP1*, we first investigated *FATP1* expression levels in the tissues of newborn and adult Qinchuan cattle. *FATP1* was widely expressed in all the tissues tested (at both ages). Expression of *FATP1* was especially high in heart, kidney, muscle, small intestine, large intestine, and perirenal fat tissues in newborn cattle. In most of the tissues investigated, including spleen, liver, reticulum, abomasum, small intestine, large intestine, muscle, kidney, and heart, *FATP1* expression decreased with age (Figure 1A). However, *FATP1* expression increased with age in omasum and perirenal fat tissues (Figure 1A). Next, we investigated *FATP1* expression levels every two days during adipocyte differentiation. *FATP1* expression was relatively stable during differentiation until the fourth day, at which time *FATP1* expression increased sharply (Figure 1B).

### 3.2. Overexpression of FATP1 Promotes Preadipocyte Differentiation in Qinchuan Cattle

**Figure 2:**
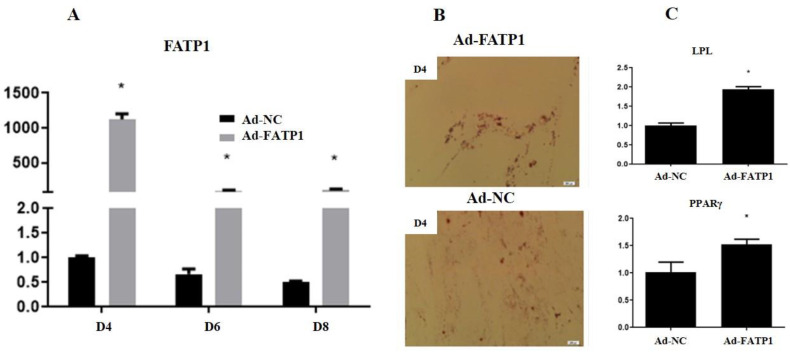
Overexpression of FATP1 promotes preadipocyte differentiation in Qinchuan cattle. (**A**) FATP1 mRNA levels in adipocytes infected with Ad-FATP1, Ad-NC at day 4 (D4), day (D6) and day 8 (D8) after differentiation (*n* = 3). (**B**) Adipocytes infected with Ad-FATP1,/Ad-NC at the 4th day of differentiation. (**C**) *LPL* and *PPARG* mRNA levels in adipocytes infected with Ad-FATP1,/Ad-NC at the 4th day after differentiation (*n* = 3). * *p* ≤ 0.05.

To further explore the role of FATP1 in preadipocyte differentiation, Ad-*FATP1* was constructed and used to infect preadipocytes (MOI, 25). The infected cells were then induced to differentiate until the 8th day. qRT-PCR analysis revealed that Ad-*FATP1* significantly promoted the expression of *FATP1* during differentiation, inducing a greater than 1100-fold increase in *FATP1* mRNA expression (in comparison with NC) by the 4th day (Figure 2A). Furthermore, Oil Red O staining provided evidence that FATP1 overexpression markedly and stably promoted lipid droplet formation (Figure 2B).

Preadipocyte differentiation into mature adipocytes is also known to involve additional marker genes. To further investigate preadipocyte differentiation, the expression levels of *PPARG*, a marker gene of adipogenic differentiation [9], and *LPL*, a marker gene of lipid metabolism [10,11], were analyzed by qRT-PCR. The results reveal that overexpression of *FATP1* up-regulated the expression of these two genes at the 4th day of differentiation (Figure 2C). Thus, overexpression of *FATP1* increased *LPL* and *PPARG* expression levels, promoted the differentiation of preadipocytes, and promoted lipid deposition in Qinchuan cattle.

### 3.3. Interference of FATP1 Expression Inhibits Preadipocyte Differentiation in Qinchuan Cattle

Three *FATP1* siRNAs were synthesized and transfected into preadipocytes, and the siRNA with the highest interference efficiency was selected for further *FATP1* knockdown experiments. All three siRNAs dramatically decreased the expression of *FATP1* (Figure S1). siRNA3 (interference efficiency, 90%) was selected for further *FATP1* knockdown experiments (and is henceforth referred to as si-*FATP1*).

**Figure 3:**
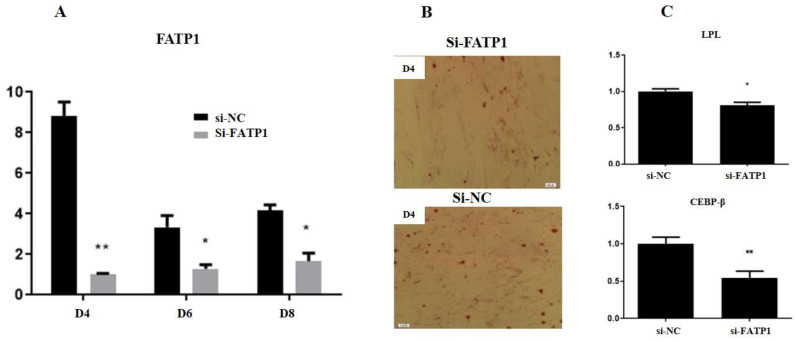
Interference of FATP1 inhibits preadipocyte differentiation in Qinchuan cattle. (**A**) mRNA levels in adipocytes infected with si-FATP1, si-NC at day 4 (D4), day (D6) and day 8 (D8) after differentiation (*n* = 3). (**B**) Adipocytes infected with si-FATP1, si-NC at the 4th day of differentiation. (**C**) *LPL* and *CEBPB* mRNA levels in adipocytes infected with si-FATP1, si-NC at the 4th day of differentiation (*n* = 3). * *p* ≤ 0.05, ** *p* ≤ 0.01.

Preadipocytes were infected with si-*FATP1* and the cells were then induced to differentiate. qRT-PCR analysis demonstrated that si-*FATP1* significantly decreased the expression of *FATP1* during adipocyte differentiation (Figure 3A). Oil red O staining revealed that knockdown of *FATP1* significantly reduced the number of lipid droplets in preadipocytes (Figure 3B). The expression levels of several adipocyte differentiation marker genes were also analyzed following interference of *FATP1*. qRT-PCR analysis demonstrates that the expression levels of *CEBPB* (encoding C/EBP-β) and *LPL* were significantly decreased following interference of *FATP1*. Thus, interference of *FATP1* inhibited the expression of *LPL* and *CEBPB*, suppressed adipocyte differentiation, and decreased lipid deposition in Qinchuan cattle.

### 3.4. Transcriptome Sequencing and Annotation of DEGs

To further explore the effects of *FATP1* gene expression on preadipocyte differentiation and lipid deposition in Qinchuan cattle, preadipocyte cells were infected/transfected with Ad-*FATP1*, Ad-NC, siRNA, and si-NC. Two days after infection/transfection, the preadipocytes were induced to differentiate. The preadipocyte cells were then harvested on the 4th day of differentiation. Each of the four treatments (Ad-*FATP1*, Ad-NC, siRNA, and si-NC) was replicated three times, producing a total of 12 samples. All 12 samples were then analyzed by RNA-Seq.

**Figure 4:**
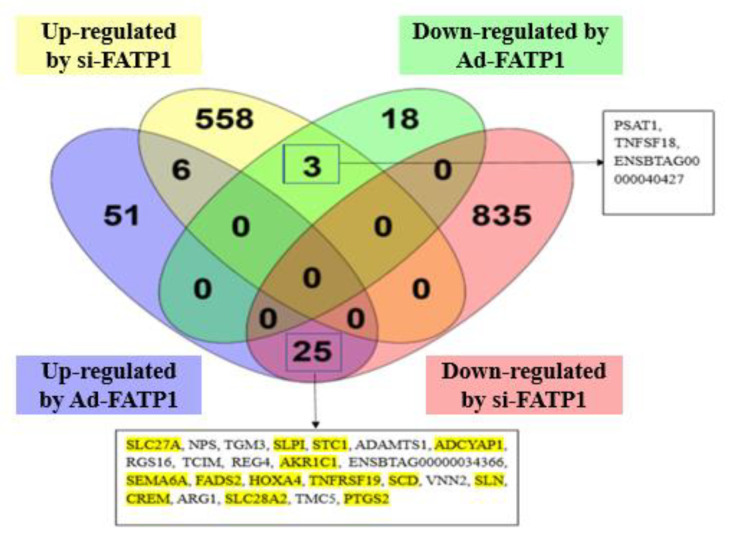
DEGs identified by RNA-seq analysis and DESeq2 following FATP1 overexpression, silencing with Ad-FATP1, Ad-NC or si-FATP1, si-NC, respectively. DEGs were selected using an adjusted *p* ≤ 0.05 and a |log_2_ (Fold Change)| ≥ 1.5. The numbers in the Venn diagram illustrate overlapping DEGs between groups. Genes related to adipocyte differentiation or lipid metabolism are shown in yellow.

**Figure 5:**
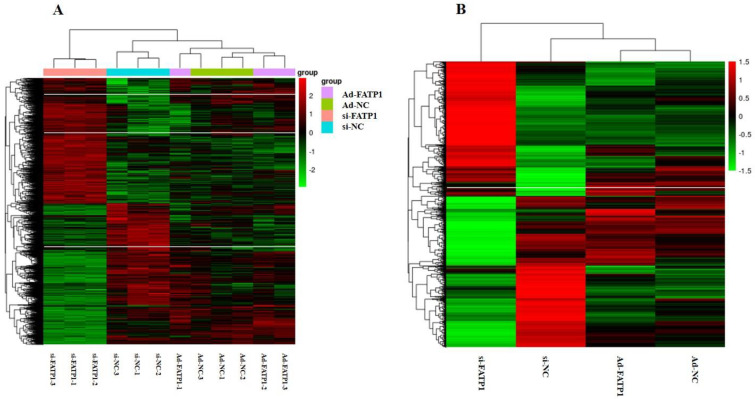
Heat maps of DEGs following FATP1 overexpression, silencing with Ad-FATP1, Ad-NC or si-FATP1, si-NC, respectively. Abscissa, sample name. Ordinate, normalized value of the differential gene (FPKM). The redder the color, the higher the expression level. The greener the color, the lower the expression level. (**A**) Clustered by individual sample. (**B**) Clustered by grouped sample.

Genes that were differentially expressed between treatments (Ad-*FATP1*, Ad-NC, siRNA, and si-NC) were identified using DESeq2. DEGs were screened using an adjusted *p* ≤ 0.05 and a |log_2_ (Fold Change)| ≥ 1.5. In total, 1496 genes were identified as DEGs. Three DEGs were up-regulated by si-*FATP1* and down-regulated by Ad-*FATP1* (*PSAT1*, *TNFSF18*, and *ENSBTAG00000040427*). In addition, twenty-five DEGs were up-regulated by Ad-*FATP1* and down-regulated by si-*FATP1* (Figure 4). Of these 28 DEGs, the functions of 14 genes were related to adipogenic differentiation and lipid deposition. To illustrate the expression patterns of DEGs in the different treatments, heatmaps were generated for individual samples (Figure 5A) and for groups of samples (Figure 5B). A comparison of Figure 5A,B reveals that the expression levels of the most dysregulated genes were consistent across all three replicates. These results also demonstrate that overexpression of *FATP1* has a greater influence on DEGs (compared with *FATP1* silencing).

**Figure 6:**
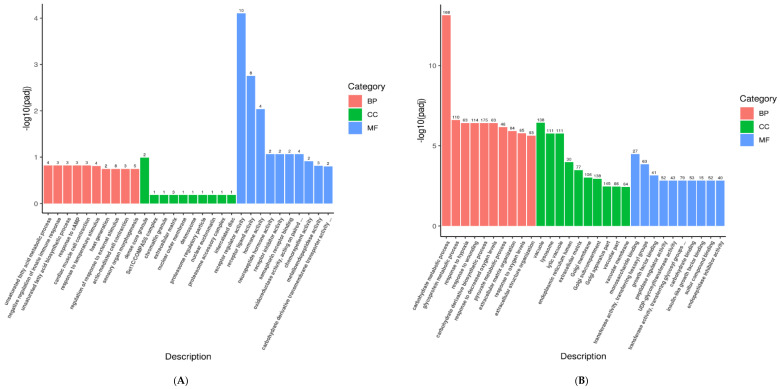
The most significant GO terms enriched within the DEGs following FATP1 overexpression, silencing with Ad-FATP1, Ad-NC or si-FATP1, si-NC, respectively. (**A**) GO terms of DEGs identified following *FATP1* overexpression. (**B**) GO terms of DEGs identified following *FATP1* interference. BP (biological processes), CC (cell components), MF (molecular functions).

**Figure 7:**
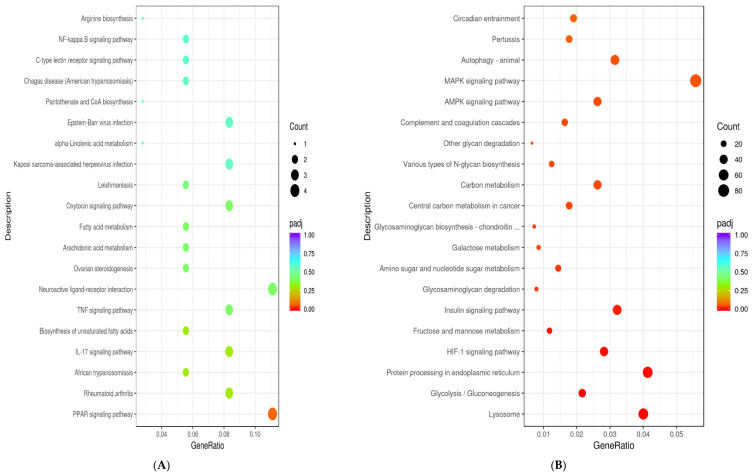
The KEGG pathways database was used to analyze pathways significantly enriched in DEGs following FATP1 overexpression/silencing with Ad-FATP1/Ad-NC or si-FATP1/si-NC, respectively. The top 20 pathways are shown. The size of each dot represents the number of genes annotated to the KEGG pathway. The color (ranging from red to purple) represents the significance of the enrichment. (**A**) The KEGG pathways of DEGs identified following FATP1 overexpression. (**B**) The KEGG pathways of DEGs identified following FATP1 interference.

**Figure 8:**
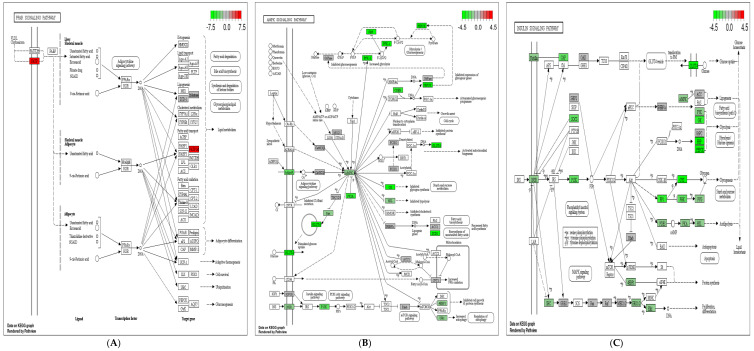
KEGG pathways enriched in DEGs following FATP1 overexpression, silencing with Ad-FATP1, Ad-NC or si-FATP1, si-NC, respectively. Red, up-regulation; Green, down-regulation. (**A**) PPAR signaling pathway DEGs in the *FATP1* overexpression group. (**B**) AMPK signal pathway DEGs in the *FATP1* interference group. (**C**) Insulin signaling pathway DEGs in the *FATP1* interference group.

To further explore the functions of DEGs, GO function enrichment analysis and KEGG pathway analysis were conducted. In the *FATP1* overexpression group, DEGs were associated with various GO terms, including unsaturated fatty acid metabolic process, dense core granule, receptor regulator activity (Figure 6A), and with the PPAR signaling pathway (Figure 7A and Figure 8A). In the *FATP1* silencing group, DEGs were associated with several different GO Terms, including carbohydrate metabolic process, extracellular structure organization, monosaccharide binding (Figure 6B), and with several pathways, including the AMPK signaling pathway and the Insulin signaling pathway (Figure 7B and Figure 8B,C).

### 3.5. Validation and Dynamic Expression of DEGs in the Adipocytes of Qinchuan Cattle

**Figure 9:**
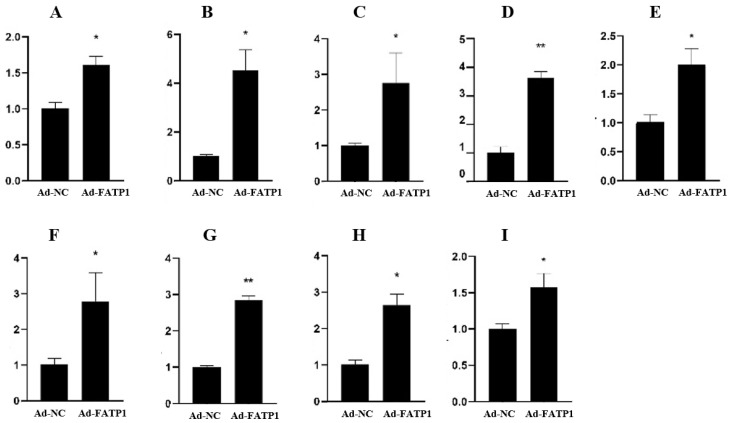
Validation of DEGs by qRT-PCR at the 4th day of differentiation in the *FATP1* overexpression group infected with Ad-FATP1, Ad-NC (*n* = 3). (**A**) *SLPI*; (B) *STC1*; (**C**) *SEMA6A*; (**D**) *TNFRSF19*; (**E**) *SLN*; (**F**) *PTGS2*; (**G**) *ADCYP1*; (**H**) *FADS2*; (**I**) *SCD*. * *p* ≤ 0.05, ** *p* ≤ 0.01.

**Figure 10:**
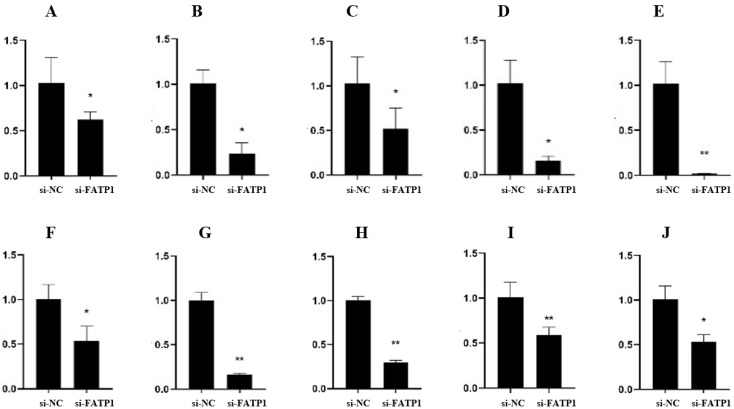
Validation of DEGs by qRT-PCR at the 4th day of differentiation in interference group infected with si-FATP1/si-NC (*n* = 3). (**A**) *SLPI*; (**B**) *STC1*; (**C**) *SEMA6A*; (**D**) *TNFRSF19*; (**E**) *SLN*; (**F**) *HOXA4*; (**G**) *ADCYP1*; (**H**) *FADS2*; (**I**) *SCD*; (**J**) *CREM*. * *p* ≤ 0.05, ** *p* ≤ 0.01.

To validate the RNA-seq and functional analysis results for DEGs, several DEGs associated with adipocyte differentiation and fat deposition were selected for further analysis. The expression levels of these genes in adipocytes treated with Ad-*FATP1*, Ad-NC, siRNA, and siNC were analyzed by qRT-PCR using *ACTB* as an internal reference gene. qRT-PCR analysis revealed that the expression levels of *SLPI*, *STC1*, *SEMA6A*, *TNFRSF19*, *SLN*, *PTGS2*, *ADCYP1*, *FADS2*, and *SCD* were significant up-regulated by overexpression of *FATP1* (Figure 9). In contrast, the expression levels of *SLPI*, *STC1*, *SEMA6A*, *TNFRSF19*, *SLN*, *HOXA4*, *ADCYP1*, *FADS2*, *SCD*, and *CREM* were significantly down-regulated by silencing of *FATP1* (Figure 10). Together, these qRT-PCR results agree with and validate the results of our RNA-seq analysis.

**Figure 11:**
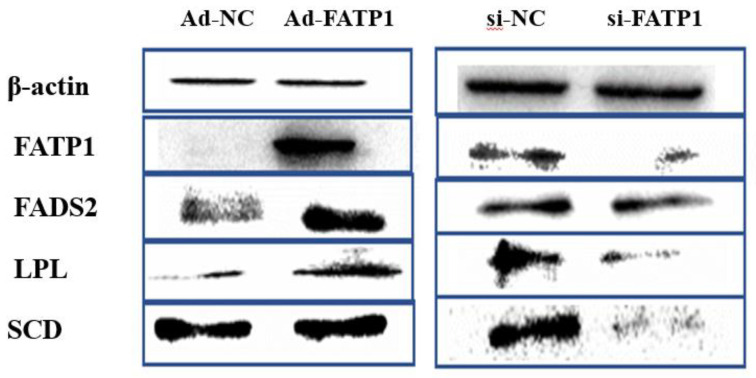
Western blotting of β-actin, FATP1, FADS2, LPL, and SCD at the 4th day of differentiation after *FATP1* overexpression and *FATP1* interference with Ad-FATP1/Ad-NC or si-FATP1/si-NC, respectively.

**Figure 12:**
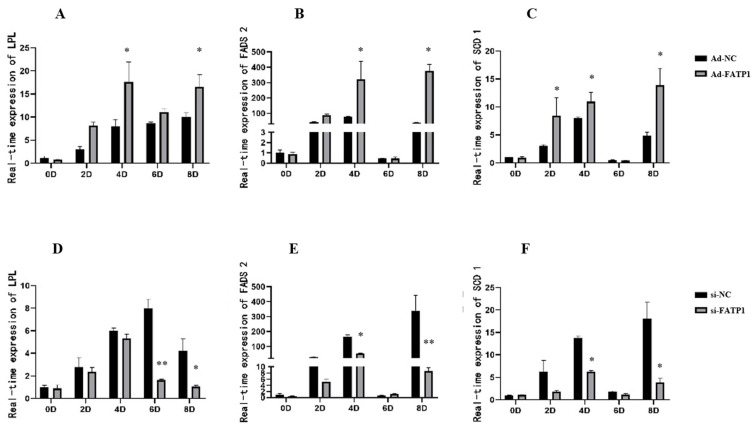
*FADS2*, *LPL*, and *SCD* expression levels during adipocyte differentiation following *FATP1* overexpression and *FATP1* interference. (**A**–**C**) *FATP1* overexpression with Ad-FATP1/Ad-NC; (**D**–**F**) *FATP1* interference with si-FATP1/si-NC. * *p* ≤ 0.05, ** *p* ≤ 0.01.

In addition, the protein expression levels of four key DEGs were investigated by western analysis following *FATP1* overexpression and *FATP1* interference in adipocytes. While *FATP1* overexpression promoted FADS2, LPL, and SCD protein levels, *FATP1* interference reduced FADS2, LPL, and SCD protein levels (Figure 11). These results are consistent with our analyses of mRNA expression levels by RNA-seq and qRT-PCR. To further understanding of the effects of *FATP1* expression levels on these proteins, we analyzed their dynamic expression during differentiation following infection,/transfection with Ad-*FATP1* or si-*FATP1*. While overexpression of *FATP1* promoted the expression of *LPL*, *FADS2*, and *SCD* during differentiation, silencing of *FATP1* inhibited their expression (Figure 12).

## 4. Discussion

In the present study, our analyses of *FATP1* expression patterns demonstrate that *FATP1* was highly expressed in heart, kidney, muscle, and adipose tissues. These results are consistent with previous reports from human and mouse studies indicating that *FATP1* is highly expressed in tissues exhibiting rapid fatty acid metabolism, including muscle, heart, and adipose tissue [3,4]. Our results also reveal that *FATP1* was highly expressed in the perirenal fat tissue of adult and infant Qinchuan cattle. Moreover, *FATP1* expression levels were demonstrated to increase during adipocyte differentiation (especially in the early stages of differentiation). The observed expression patterns of *FATP1* in fat tissue and during adipocytic differentiation indicate that FATP1 may play a crucial role in fat formation in Qinchuan cattle.

To further elucidate the function of FATP1 during adipogenesis in Qinchuan cattle, overexpression and interference assays were employed. Although FATP1 gain,/loss of function could be demonstrated following *FATP1* overexpression,/interference, FATP1 was observed to play a role in adipocyte differentiation under both assay conditions. *FATP1* overexpression was demonstrated to increase deposition of lipid droplets and to up-regulate the expression of lipid metabolism-related genes, including *LPL* and *PPARG*. In contrast, *FATP1* interference was demonstrated to decrease deposition of lipid droplets and to down-regulate expression of lipid metabolism-related genes. Together, these results provide evidence that the *FATP1* gene plays a regulatory role in adipogenic differentiation and lipid deposition in Qinchuan cattle. This conclusion is consistent with previous research on porcine intramuscular preadipocytes and chicken preadipocytes [10,11].

To unravel the role of FATP1 in adipocyte differentiation, RNA-Seq analysis was conducted on *FATP1* overexpression,/interference cell models. Using a |log_2_ (Fold Change)| ≥ 1.5 and a *p* ≤ 0.05 as screening conditions, DEGs in the overexpression and interference groups were identified. These DEGs were then sorted into an up-regulated gene set and a down-regulated gene set. In addition, we identified the gene set up-regulated by Ad-*FATP1* and down-regulated by si-*FATP1*, and the gene set up-regulated by si-*FATP1* and down-regulated by Ad-*FATP1*. In total, 28 genes in these two gene sets were identified. Finally, we identified all the genes within these two gene sets whose functions are related to adipogenic differentiation and fat deposition. These were observed to include *SLPI*, *STC1*, *EMA6A*, *TNFRSF19*, *SLN*, *PTGS2*, *ADCYP1*, *FADS2*, *SCD*, *HOXA4*, and *CREM*.

SLPI (secretory leukocyte protease inhibitor) is known to suppress proinflammatory responses through inhibition of NF-κB signaling, and also to demonstrate immuno-modulatory activities on inflammatory diseases [14]. In addition, SLPI has been demonstrated to play a role in obesity, with one study reporting that SLPI constrained obesity by increasing browning—SLPI levels were positively correlated with browning and negatively correlated with obesity [15].

Tumor necrosis factor receptor superfamily (TNFRSF) members are glycoproteins with a single transmembrane region, a cysteine-rich extracellular ligand binding domain, and a diverse cytoplasmic tail. TNFRSF19 is up-regulated by the Wnt signaling pathway and down-regulated by C/EBP. TNFRSF19 is known to inhibit adipogenic differentiation and thus function as a negative regulator of adipocyte differentiation. *TNFRSF19* overexpression,/knockdown was also observed to significantly decrease,/increase adipogenesis [16].

ADCYAP1 is known to play roles in glucose and energy homeostasis [17], and in the regulation of lipid metabolism [18]. In an *ADCYAP1* knockout mouse model, white adipose tissue (e.g., epididymal, retroperitoneal, and abdominal subcutaneous adipose tissues) was significantly reduced in size and mass. In addition, the expression levels of *AP2* (encoding the fatty acid binding protein aP2), an important marker gene for adipocyte differentiation, were decreased after *ADCYAP1* knockout. The results obtained with the *ADCYAP1* knockout mouse model demonstrate the important role played by ADCYAP1 in lipid metabolism and regulation of body weight [19].

Fatty acid desaturase 2 (FADS2) is a member of the fatty acid desaturase protein family. FADS2 catalyzes the desaturation reaction during long chain (≥C20) polyunsaturated fatty acid (LC-PUFA) synthesis, and is pivotal in LC-PUFA biosynthesis and maintaining LC-PUFA homeostasis [20]. Genetic studies have revealed that a polymorphism in the *FADS2* gene affects the fatty acid composition of bovine milk by modifying mir-744 binding [21,22]. In addition, PUFAs were found to have a direct effect on *FADS2* expression in differentiated 3T3-L1 adipocytes [23]. Research on the mechanism of action of FADS2 in human and mouse found that *FADS2* was the target of mTOR and SREBP signaling. While activation of the mTORC1 signaling pathway increased *FADS2* expression, inhibition reduced *FADS2* expression. Likewise, overexpression of SREBP-1/2 increased *FADS2* expression [24]. SREBP-1 and PPARα were both demonstrated to up-regulate *FADS2* promoter activity in a Luciferase reporter assay [25].

Stearoyl-CoA Desaturase 1 (SCD1) is a key regulator of de novo lipogenesis, acting as a rate-limiting enzyme. SCD1 catalyzes the synthesis of MUFAs. Loss of SCD1 potentiates beige adipocyte formation in mice and SCD1 deficiency can induce adipose-derived mesenchymal stem cells to differentiate into beige adipocytes [26]. Overexpression of SCD1 in the subcutaneous adipose tissues of mouse revealed that SCD1 can upregulate lipases and induce lipolysis, further promoting energy expenditure and fat mobilization. Furthermore, *SCD1* knockdown inhibited lipolysis in adipocytes by down-regulating lipases and reducing lipophagy. BMP4 was also demonstrated to be a crucial factor regulating *SCD1* expression, up-regulating *SCD1* expression through the Smad pathway [27].

Lipoprotein lipase (LPL) is a multifunctional enzyme that plays a pivotal role in lipid transport and metabolism [28], especially in the hydrolysis of triglycerides [29]. *LPL* expression is known to be regulated in a tissue-specific manner. Moreover, nutrient levels and hormonal states also have a profound effect on *LPL* expression. *LPL* is also an important marker gene for adipocyte differentiation, and *LPL* expression levels increase with triglyceride accumulation during preadipocyte differentiation [30]. In addition, LPL is essential for fatty acid storage and uptake during 3T3-L1 preadipocyte differentiation [31]. When *LPL* expression is inhibited, the absorption of exogenous lipids is hindered, thus affecting the deposition of fat [32].

Taken together, the research described above provides evidence that the aforementioned genes are involved in fat deposition and fatty acid metabolism directly or indirectly. In the present study, we used qRT-PCR to validate the effects of FATP1 on the expression of these genes through *FATP1* overexpression and *FATP1* silencing. These results are also consistent with our RNA-seq results, providing further support for the view that FATP1 influences the expression of key genes and regulates adipocyte differentiation and fatty acid metabolism. Additional research is required to explore the mechanism of action by which FATP1 regulates these adipogenic genes.

## 5. Conclusions

In summary, we demonstrate that *FATP1* is extensively expressed in the tissues and differentiating adipocytes of Qinchuan cattle. While *FATP1* overexpression promoted preadipocyte differentiation in Qinchuan cattle, *FATP1* interference demonstrated the opposite effect. Through RNA-seq analysis of adipocytes in which *FATP1* was overexpressed,/silenced, we were able to identify DEGs related to fat metabolism. The genes in this set included *SLPI*, *STC1*, *SEMA6A*, *TNFRSF19*, *SLN*, *PTGS2*, *ADCYP1*, *FADS2*, and *SCD*. Functional annotation revealed that these DEGs were enriched in the PPAR signaling pathway, AMPK signal pathway, and Insulin signaling pathway. These findings provide evidence that *FATP1* is a candidate gene for the regulation of fat deposition and fatty acid metabolism in cattle and might devote to beef cattle breeding.

## Figures and Tables

**Table 1:** The sequences of siRNAs for interference of bovine FATP1.

siRNA Name	Sequence
siRNA1 siRNA2 siRNA3 siNC	5′-3′ CCAACGCUGUGGCCAACUUTT 5′-3′ AAGUUGGCCACAGCGUUGGTT 5′-3′ GCAUGGAUGAUCGACUCUUTT 5′-3′ AAGAGUCGAUCAUCCAUGCTT 5′-3′ GCGUGGGUCAGUGUCUCAUT 5′-3′ AUGAGACACUGAACGCCCCTT 5′-3′ ACGUUUCUCCGAGCACGUTT 5′-3′ CGUUACGUGACACGGAGAATT

**Table 2:** The primer sequences for qRT-PCR.

Primer	Sequence (5′-3′)	Fragment Size (bp)
FATP1-F	AAGAGCCTGGTCAACTG	186
FATP1-R	TAGGAGTAGTGCCCTGC	
β-actin-F	CATCAATGAGCGTCGGTTCC	147
β-actin-R	CCGTGGCGTAGCAGAGGTC	
PPAR-γ-F	GAGATGAGTACACACGCCAAG	216
PPAR-γ-R	GGGCTAAAGTCCATCACCAA	
LPL-F	GATGATGCGGATTTTGTAGACG	156
LPL-R	GTTGGAAAGTGCCTCCGTTAG	
C/EBP-β-F	TTCCTCTCCGACCTCTTCTC	173
C/EBP-β-R	CCAGACTCACGTAGCCGTACT	
SLPI-F	AGACGACCAGTGCGTAGGTA	143
SLPI-R	TCCCAAACGGCAGGAATCAG	
STC1-F	AGTGATTCGCTGCCTCAACA	107
STC1-R	CTCCTGGACTTCGGCAATCA	
AKR1C1-F	TCGATGGCCTCAACAGCAAT	186
AKR1C1-R	TGGGGAAGACAAGAATCAATGC	
SEMA6A-F	GGGCTGCTTTCCCAGAAGAT	209
SEMA6A-R	TATGGTCCCTGGCAGCAATG	
HOXA4-F	AGAAGATCCATGTCAGCGCC	194
HOXA4-R	CGGGTCAGGTAGCGGTTAAA	
TNFRSF19-F	GCTAGTGAGGAGCCGACCC	174
TNFRSF19-R	TCTGTTGCCTGAGTAGCACG	
SLN-F	CAGCCCAGTGTGTCCTTGAC	216
SLN-R	AGGCAAACTTCTGAGGGCAC	
PTGS2-F	TTGGCTACGGGAACACAACA	159
PTGS2-R	AAGGGGATGCCAGTGGTAGA	

## Supplementary Materials

The following are available online at https://www.mdpi.com/article/10.3390/ani11102789/s1, Figure S1. FATP1 mRNA levels in adipocytes infected with siRNA1/siRNA2/siRNA3, and si-NC (*n* = 3).
