# Supplementary figures and images for: The Effect of FATP1 on Adipocyte Differentiation in Qinchuan Beef Cattle
Source: Animals (Basel). 2021 Sep 24;11(10):2789. doi: 10.3390/ani11102789 (PMC8532991; doi:10.3390/ani11102789)

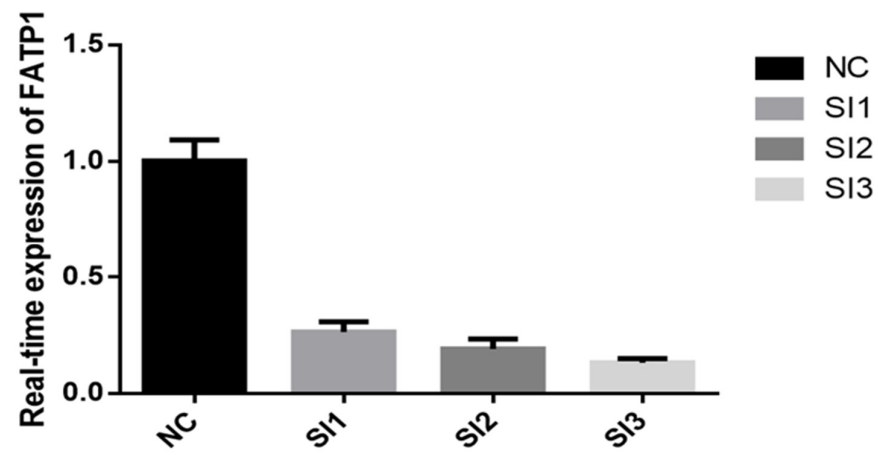

**Figure S1.** *FATP1* mRNA levels in adipocytes infected with siRNA1/ siRNA2/ siRNA3/ and si-NC (n=3).

Supplement: Supplementary file 1 [file animals-11-02789-s001.zip › animals-1361175-supplementary.pdf]
